# The changing landscape of cerebral revascularization surgery: A United Kingdom experience

**DOI:** 10.3389/fradi.2022.981501

**Published:** 2022-09-08

**Authors:** Mathew J. Gallagher, Joseph Frantzias, Ahilan Kailaya-Vasan, Thomas C. Booth, Christos M. Tolias

**Affiliations:** Kings College Hospital, London, United Kingdom

**Keywords:** cerebral bypass surgery, cerebral bypass, cerebral revascularisation, cerebral revascularization surgery, flow diversion, flow diverting stent, chronological, centralization

## Abstract

**Objective:**

We describe the chronological trends in cerebral revascularization surgery through a single-surgeon experience; and we review whether in the context of giant and fusiform cerebral aneurysms, flow-diverting stents have impacted on the use of cerebral revascularization surgery.

**Methods:**

We review our single institution prospectively collected database of cerebral revascularization procedures between 2006 and 2018. Comparing this to our database of flow-diverting endovascular stent procedures, we compare the treatment of fusiform and giant aneurysms. We describe patient demographics, procedural incidence, complications, and outcomes.

**Results:**

Between 2006 and 2018, 50 cerebral revascularization procedures were performed. The incidence of cerebral revascularization surgery is declining. In the context of giant/fusiform aneurysm treatment, the decline in cerebral revascularization is accompanied by a rise in the use of flow-diverting endovascular stents. Thirty cerebral revascularizations were performed for moyamoya disease and 11 for giant/fusiform aneurysm. Four (14%) direct bypass grafts occluded without neurological sequela. Other morbidity included hydrocephalus (2%), transient ischemic attacks (2%), and ischemic stroke (2%). There was one procedure-related mortality (2%). Flow-diverting stents were inserted for seven fusiform and seven giant aneurysms. Comparing the treatment of giant/fusiform aneurysms, there was no significant difference in morbidity and mortality between cerebral revascularization and flow-diverting endovascular stents.

**Conclusion:**

We conclude that with the decline in the incidence of cerebral revascularization surgery, there is a need for centralization of services to allow high standards and outcomes to be maintained.

## Introduction

Yasargil and Yonekawa (1) first described the original cerebral revascularization technique in 1977 using the superficial temporal artery. The second-generation iteration using grafts enabled the technique to develop intracranial-to-intracranial anastomosis and apply to a broader group of conditions. By the turn of the millennium, the indications for revascularization had grown but its ascendance was short-lived (2). In 2012, the early termination of the Carotid Occlusion Surgery Study and the recent United States Food and Drug Administration approval of a new endovascular flow-diverting device called the Pipeline Embolization Device (3) questioned the utility of cerebral revascularization.

In this study, we review our single-surgeon experience of cerebral revascularization surgery from 2006 to 2018 and explore how this should influence future training and commissioning of this service. We will review our treatment of giant and fusiform aneurysms from 2006 to 2018 to see what impact the introduction of flow-diverting endovascular stents has had on the utilization of cerebral revascularization surgery.

## Materials and methods

Our prospectively collected database of cerebral revascularization and endovascular procedures was reviewed for the period of May 2006 to January 2018. We confirmed this with the electronic patient records (Allscripts Sunrise, Chicago, Illinois) and individual surgeon's databases. The study did not require approval by our institutional or regional ethics committee.

Patient demographics and operative data were collected. We stratified revascularization operations and endovascular flow-diverting stent insertion by indication and surgical technique. Complications after revascularization and stent insertion were also quantified at the point of last follow-up. We assessed cerebral bypass patency in the first 24 h post-procedure, at 6 months, and between 24 and 48 months post-procedure with either computed tomography intracranial angiogram (GE Optima 660, Voxel 0.625 80KV, 315mA, Pitch 0.984:1, Noise index 9.0) or catheter intracranial angiogram (Biplane angiography, Allura Xper FD, Philips Healthcare, Amsterdam, Netherlands). In the case of cerebral aneurysm, there was no treatment protocol but endovascular treatment was favored where feasible.

We assessed aneurysm occlusion post-stent insertion on TOF MRA imaging (Signa 1.5 T HDX; GE Healthcare; or AERA 1.5T, Siemens, Erlangen, Germany). Adequate occlusion was graded as Raymond scale 1&2.

Data are presented with a median and range for continuous variables and proportions for categorical variables. For treatment-associated morbidity and mortality, results were compared using Fisher's exact test to give significance calculated on GraphPad. Significance was *p* < 0.05.

## Results

Between 2006 and 2018, 50 cerebral revascularization procedures in 43 patients were performed. Twenty-nine of these were direct bypass procedures of which 19 were STA-MCA, 8 were EC-IC, one M2-M2, and one A2-A2 bypass. Seven of the 29 procedures were performed with the excimer laser non-occlusive anastomosis (ELANA) technique. Our experience with the ELANA technique is published elsewhere (4). Of the 21 indirect bypasses, all were performed using the encephalo-duro-arterio-synangiosis (EDAS) technique. The patient median age was 34 (range 8–76) years, and 24 (56%) were female. Thirty procedures were performed for moyamoya disease, and 11 cases were performed for aneurysm occlusion. Eight procedures were performed for carotid artery stenosis prior to 2012, and 1 bypass was performed for skull base tumor. Demographics are detailed in Table 1.

**Table 1:** Demographics of cerebral revascularization cases.

**Cerebral revascularisation**	**Number (%)**
Years	12
Number treated	50
Median age (range) years	34 (8–76)
Sex M:F	19: 24
**Technique:**	
Direct bypass Indirect EDAS	29 21
**Indication:**	
Moyamoya Aneurysm Carotid stenosis Skull base tumor	30 (60%) 11 (22%) 8 (16%) 1 (2%)
**Morbidity:**	
Direct bypass graft occlusion Hydrocephalus Transient ischaemic attack Other neurological deficit Stroke Procedure related mortality	4 (14%) 1 (2%) 1 (2%) 1 (2%) 1(2%)

In total, four cerebral bypass grafts occluded, one STA-MCA graft and three EC-IC interposition grafts. None of these occlusions resulted in neurological deficit. All occurred in the first 6 months postoperatively. It is of note that three of the occlusions occurred in 2006 during our early experience with the procedure. Other significant morbidity included one (2%) delayed presentation 3 months post-procedure with symptomatic hydrocephalus requiring ventriculo-peritoneal shunt. One (2%) patient suffered transient ischemic attacks several months after surgery when he stopped taking clopidogrel. One (2%) patient suffered an ischemic stroke with hemiparesis thought to be secondary to MCA perforator occlusion in the immediate postoperative period. There were two mortalities, of which one (2%) was procedure-related. This patient suffered a malignant ICA ischemic infarct with carotid dissection, thought to be secondary to the proximal anastomosis.

**Figure 1:**
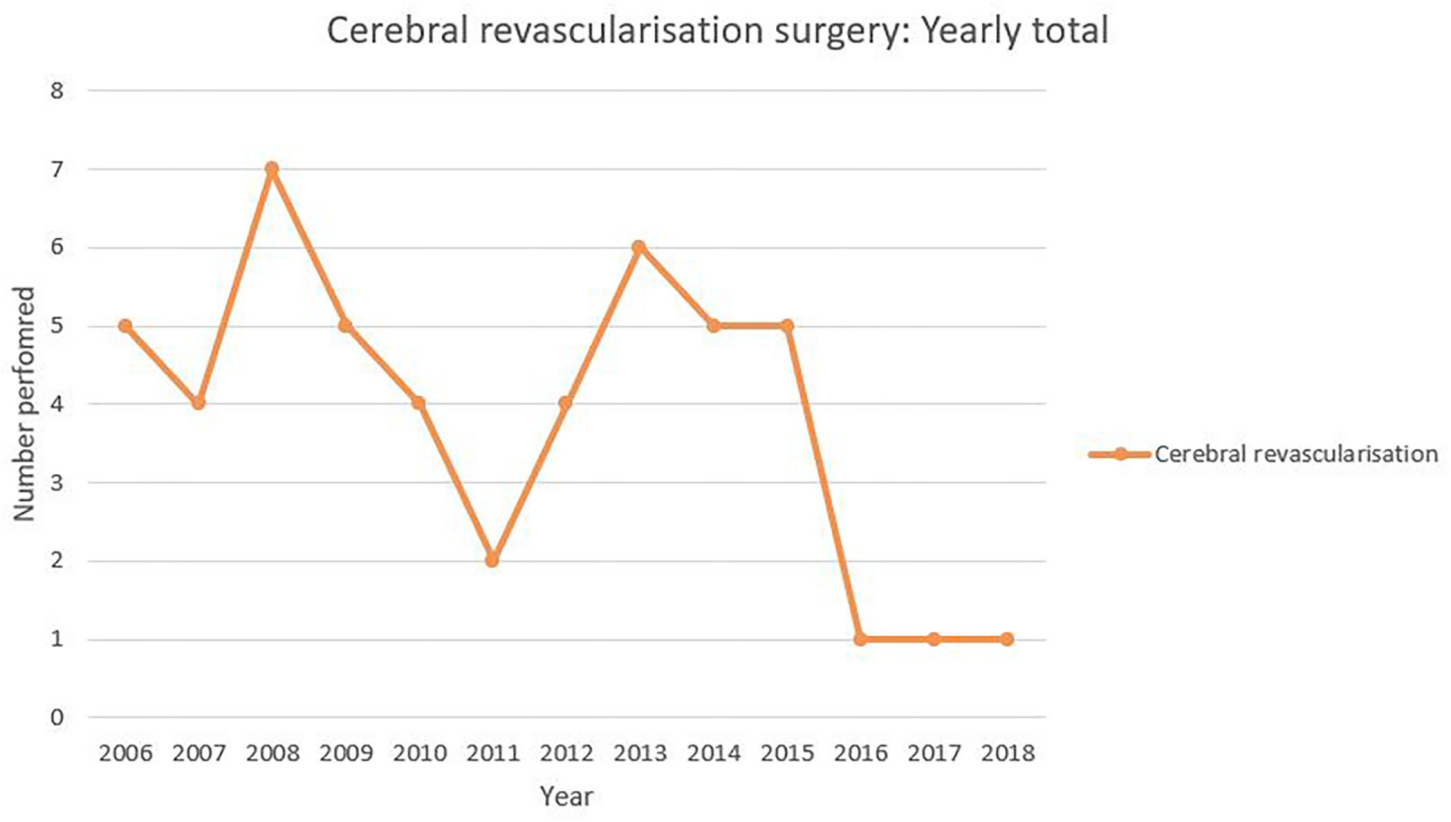
Yearly incidence of cerebral revascularization surgery.

The procedure incidence in 12-month periods is shown in Figure 1. The maximum of seven cerebral revascularization procedures performed in 2008 with a reduction down to one performed per year for the last 3 years.

Examining specifically the treatment of complex and giant aneurysms (Table 2), the data show that there were 13 patients with attempted cerebral revascularization procedures. Two procedures were abandoned due to technical difficulties: in one case due to the arachnoid adhesions from previous subarachnoid hemorrhage and in the second due to vessel atherosclerosis. Of the 11 completed cerebral revascularizations, eight (62%) were female and the median age was 51 years (range 45–68). Ten were anterior circulation aneurysms, and three had been previously treated with two previous clippings and one previous endovascular treatment. Six used the ELANA technique. There were five EC-IC bypass, four STA-MCA bypass, one M2 to M2 side to side, and one A2 to A2 side to side bypass. On follow-up, there were two bypasses with graft occlusion but adequate aneurysm occlusion and no new focal neurological deficit. Complications included one EC-IC bypass that developed a postoperative hemiparesis and one STA-MCA bypass that developed postoperative hydrocephalus and required a VP shunt, and there was one mortality in a STA-MCA bypass that had an on-table aneurysm rupture after successful bypass and had a subsequent MCA stroke.

**Table 2:** Treatment of giant/fusiform aneurysm demographics and treatment morbidity.

	**Cerebral revascularisation (%)**	**Endovascular flow diversion (%)**	**p-value**
Number treated	11 (2 abandoned)	14	
Median age (range)	51 (45-68)	57 (39-76)	
Sex M:F	3:8	4:10	
Anterior circulation	10/11	12/14	
**Previous treatment:**			
Surgical clipping	2 (18%)	0	
Endovascular	1 (9%)	4 (29%)	
Graft occlusion at f/u	2 (18%)		
Delayed aneurysm occlusion		4 (29%)	
Stent retreatment		1 (7%)	
**Morbidity:**			
Hydrocephalus	1 (9%)	0	0.44
Cranial nerve deficit	0	1 (7%)	1.0
Stroke	1 (9%)	1 (7%)	1.0
Procedure related mortality	1 (9%)	2 (14%)	1.0

Between 2009 and 2018, there were 121 flow-diverting stents inserted for the treatment of aneurysms in 113 patients. Eighty-eight (77.9%) patients were female, and the median age was 55 (range 16–76). The treated aneurysms had wide necks (neck > 4 mm) in 90/121 (74.4%) cases, and seven (5.8%) were fusiform. Fifty-four (44.6%) were large aneurysms (10–25 mm), and 7 (5.8%) were giant aneurysms. Four (3.3%) stents required retreatment, and in 4 (3.3%) cases, non-delivery occurred, due to anatomical restrictions. In the period 2009 to 2018, there were 14 giant or fusiform aneurysms treated endovascularly with flow-diverting stents. Ten were female, and the median age was 57 years (range 39–76). Twelve were anterior circulation aneurysms, and four were recurrent aneurysms after previous endovascular coiling. There were four aneurysms with delayed occlusion after endovascular flow diversion. Two of these became occluded at 18-month follow-up, one at 24 months and one at 30 months. One patient, a recurrent right communicating segment ICA wide-necked fusiform aneurysm, required retreatment after 5 months with a further flow-diverting stent. Two patients suffered significant morbidity, and there were two mortalities. One patient developed an ipsilateral third nerve palsy and remote intracerebral hematoma after treatment of a 28 mm right cavernous segment aneurysm with 2 flow-diverting stents; a second patient treated for a mid-basilar fusiform aneurysm suffered a small pontine infarct with right hemiparesis and dysarthria. There were two mortalities in this group. One previously endovascular coiled giant basilar aneurysm (27 mm) treated with a single stent suffered an acute subdural hematoma 15 days after treatment and passed away. A second giant terminal segment ICA aneurysm (30 mm) suffered an acute aneurysmal rupture 2 days after successful stent deployment.

**Figure 2:**
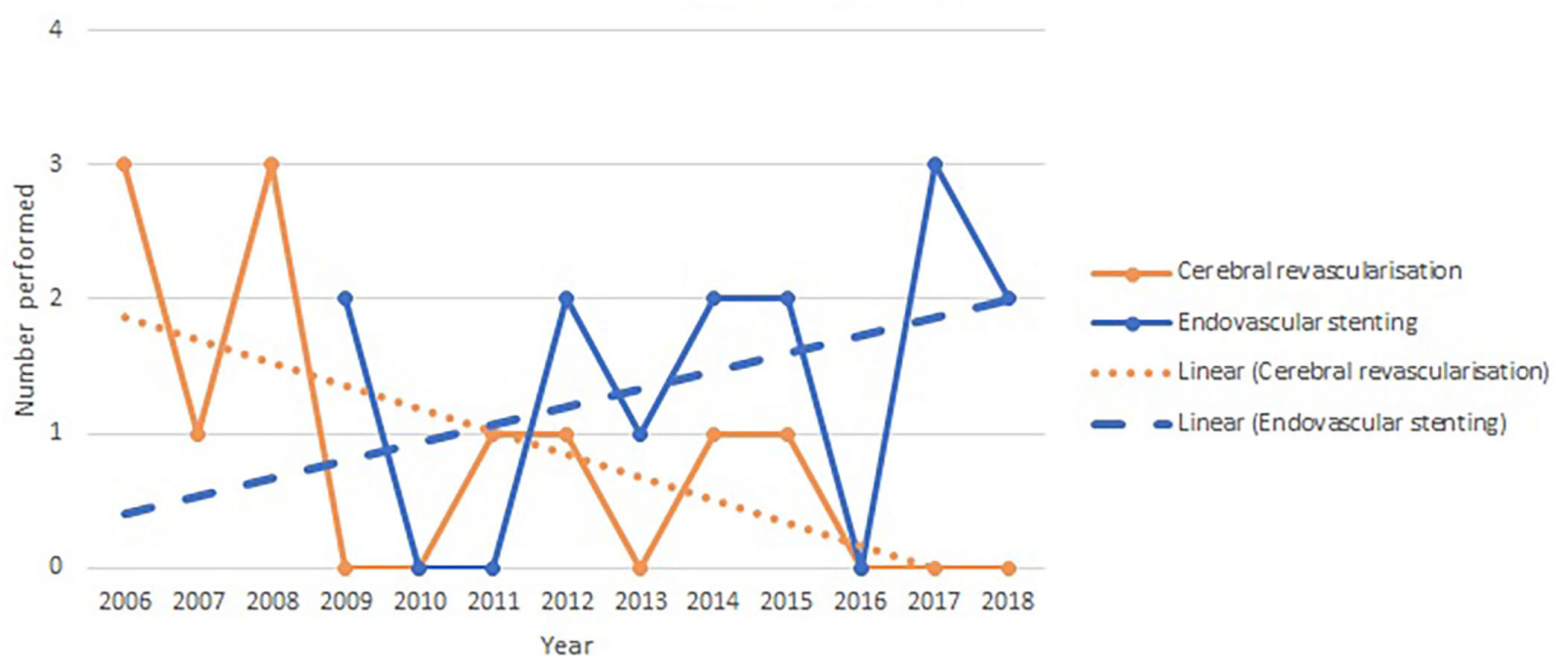
Yearly incidence of cerebral revascularization and endovascular flow diversion surgery for giant and fusiform aneurysms.

The yearly incidence of cerebral revascularization and endovascular flow diversion procedures for giant or fusiform aneurysms is shown in Figure 2. There is a trajectory of increasing use with a positive line of best fit (R^2^ value 0.14) for endovascular flow diversion and a decline in use and a negative line of best fit (R^2^ value 0.38) for cerebral revascularization surgery.

## Discussion

We present the only published cohort of modern cerebral revascularization procedures performed in the United Kingdom. However, in our center with a dedicated neurovascular surgical service, the incidence of these procedures is declining.

Yasargil developed the EC-IC bypass from the STA to the MCA with the first procedure in a human in 1969 (1). As the technique developed through the 1980s, the list of indications grew to include occlusive ischemic disease, giant and or fusiform aneurysms, vertebrobasilar insufficiency, multi-infarct dementia, and ischemic retinopathy (2). However, the failure of the EC-IC bypass study (5) to show reduction in stroke or stroke-related death compared to best medical therapy in patients with carotid or MCA stenosis and recent stroke led neurosurgeons to question its efficacy. With an evolved design and more selected patient group, the randomized Carotid Occlusion Surgery Study in 2012 (6) again failed to demonstrate an improvement in 2-year stroke incidence when comparing STA-MCA bypass with best medical therapy in patients with complete ICA occlusion. This reduced the indications for cerebral revascularization procedures to giant/fusiform aneurysms, moyamoya disease, and skull base tumors.

The approval of endovascular flow-diverting stents in 2011 has changed (particularly in Europe) how we approach giant and complex aneurysm treatment. Through endoluminal reconstruction and remodeling of the parent artery, they are an effective treatment (7). The extrapolation of treatment outcome and safety data from ISAT (8) in part has led endovascular techniques to be seen as the primary treatment choice. There is no Class 1 evidence comparing endovascular treatment with surgical treatment for giant/fusiform aneurysms. A meta-analysis of cohort studies shows similar rates of morbidity and mortality with the two treatment modalities (9). Although our two groups are not equally weighted and matched for aneurysm size and location, we observed a non-significant increased rate of mortality (14% vs. 9%) in the endovascular treatment group compared to the cerebral revascularization group. This finding has been replicated in an American cohort where the risk of mortality in unruptured giant aneurysms was higher in the endovascular vs. surgical treatment group (12% vs. 3%) (10).

We show in Figure 1 that the utilization of cerebral revascularization procedures is declining over time. In the context of giant and fusiform aneurysm (Figure 2), this decline is seen with an increasing use of endovascular flow-diverting stents. There is also the significant decrease in cerebral revascularization as a result of the failure of COSS study (6). It appears clear from the data that in our practice, the indication for the majority of bypass cases going forward will be moyamoya disease. This is a shift mirrored around the world. Straus et al. (11) demonstrated not only that the use of cerebral revascularization declined after introduction of flow-diverting stents but also that the patients selected for cerebral revascularization had a poorer pre-operative modified Rankin score. Lawton's personal series (12) also demonstrated a reduction in cases of cerebral revascularization for aneurysm after FDA approval of the flow diverter stent and with it an increase in the use of cerebral revascularization as a rescue procedure after failed endovascular stenting (10.8% of aneurysms treated with cerebral revascularization). Despite this decline, the Barrow is one of very few institutions to show an increase in the overall yearly incidence of cerebral revascularization (13). The rest of the neurovascular community may struggle to replicate the Barrow's large case series and success rates. With a focus on evidence-based decision-making and the prospect of medico-legal challenges when this is not apparent, other centers will struggle to adopt new revascularization techniques and follow the Barrow's lead. Therefore, a reduction in cerebral revascularization incidence is inevitable.

In this new era of reduced case load and increased complexity, where cerebral revascularization surgery for aneurysms is in part a rescue procedure, we believe that we need to address the question of how to maintain the required skill set among neurosurgeons and the rest of the surgical team. A recent analysis of EC-IC bypass in the United States showed that high-volume centers performing more than 10 cases per year resulted in reduced rates of ischemic stroke and readmission (14). Although in our study the infrequent case-related morbidity could not be correlated with yearly incidence, the impact of institutional case volume in aneurysm treatment has been explored. We showed that United Kingdom centers treating more aneurysmal subarachnoid hemorrhage patients with open surgery have better overall outcomes for endovascular and surgical intervention (15). With unruptured aneurysm treatment, Barker et al. show high-volume institutions have lower morbidity and mortality (16). The influence of surgeon experience on outcomes for unruptured aneurysm surgery and following intraoperative aneurysmal rupture shows that higher surgical numbers lead to better outcome (16–18). Analysis of microvascular decompression surgery in the United States demonstrated lower morbidity in high-volume centers with high case volume surgeons (19). In pediatric neuro-oncology surgery, it has become accepted that centralization to increase the numbers treated in each center improves results (20, 21). This movement to centralization has been adopted in neurovascular services in Norway (22) and has been previously suggested in the United Kingdom for cerebral revascularization surgery (23). Although not formalized, neurovascular surgery in the United States has in effect become centralized with aneurysm surgery being concentrated in fewer centers (24) and a reversion to the era of Charles Drake where vertebrobasilar aneurysms were transferred to specialist centers (25). We propose that in the United Kingdom and therefore most European countries, we should work toward a formal centralization of cerebral revascularization surgery to maintain high standards and good outcomes.

This study is limited as a single-center review. There is not a national database of cerebral revascularization surgery in the United Kingdom. A strict treatment choice protocol was not followed but the study represents a pragmatic review of clinical practice. The statistical use of R-squared measure of model fit for linear regression is limited by the small incidence of giant/fusiform aneurysm treatment. Long-term follow-up is not part of our prospective database but would be beneficial.

We observed a reduction in the incidence of neurovascular bypass surgery. However, there remains a clear indication for the use of bypass surgery. In the case of giant/fusiform aneurysms, the decline in cerebral revascularization surgery correlates with an increase in the utilization of endovascular flow-diverting stents. We propose that to maintain a high level of surgical competency and excellent neurological outcomes, the neurovascular bypass surgical service in the United Kingdom should be formally centralized.

## Data availability statement

The raw data supporting the conclusions of this article will be made available by the authors, without undue reservation if governance and ethical approvals are obtained.

## Ethics statement

Ethical review and approval was not required for the study on human participants in accordance with the local legislation and institutional requirements. Written informed consent for participation was not required for this study in accordance with the national legislation and the institutional requirements.

## Author contributions

CT conceived paper. MG and JF collected data and wrote paper. AK-V, TB, and CT contributed to paper and reviewed paper. All authors contributed to the article and approved the submitted version.

## Funding

This research was funded in part, by the Wellcome Trust (WT203148/Z/16/Z). For the purpose of open access, the authors have applied a CC BY public copyright licence to any Author Accepted Manuscript version arising from this submission.

## Conflict of interest

Speakers Bureau for Medtronic United Kingdom: TB; Core laboratory for Microvention: TB; Wellcome/Engineering and Physical Sciences Research Council Center for Medical Engineering (WT 203148/Z/16/Z): TB. The remaining authors declare that the research was conducted in the absence of any commercial or financial relationships that could be construed as a potential conflict of interest.

## Publisher's note

All claims expressed in this article are solely those of the authors and do not necessarily represent those of their affiliated organizations, or those of the publisher, the editors and the reviewers. Any product that may be evaluated in this article, or claim that may be made by its manufacturer, is not guaranteed or endorsed by the publisher.
